# Nervous Lesions in Amyotrophia of Articular Origin

**Published:** 1903-05-23

**Authors:** 


### NERVOUS LESIONS IN AMYOTROPHIA OF ARTICULAR ORIGIN.

In a paper published in the *Journal of Mental Pathology*, Dr. G. Pighini records the results of experiments which he had undertaken to investigate the pathology of muscular atrophy following on joint affections. Vulpian originated the theory that the muscular atrophy was due to nerve influence and was a reflex action. Deroche, Raymond, and Hoffa demonstrated the value of this theory, and moreover showed that the atrophy could be stopped by severing the continuity of the reflex arc. Dr. Pighini's experiments, which were performed on rabbits, had for their special object to determine whether or not any changes occurred in the nerve structures forming the reflex arc which could be demonstrable under the microscope. Various chemical substances and bacterial cultures were injected into the joints and the effects carefully observed. The most striking and rapid results were obtained in the joints injected with irritating chemical substances, sepsis being carefully avoided. The muscles most markedly affected in all cases were the extensors above the affected joint, and atrophy of these invariably occurred when an arthritis had been brought about by any of the means employed. In the case of an injection into the elbow-joint the triceps was most affected, and in arthritis of the knee atrophy of the quadriceps and adductor muscles ensued. In relation to the sound limbs the atrophied ones lost from one-fifth to one-third of the normal weight. The rapidity and gravity of the developed amyotrophia appeared to be directly dependent upon the irritative qualities of the pathogenic agent. The atrophy was preceded by contraction of the affected muscles and was accompanied or followed by paresis. Histological examinations showed lesions in the nerves supplying the injured joint, in the corresponding spinal ganglia and in the spinal cord, as well as in the muscles. The nerves showed a periaxile neuritis, the myeline being completely absent in some parts and simply wasted in others. True degeneration was not found. In the spinal ganglia and spinal cord the nerve cells were found to be affected in varying degree from simple deposits in the nucleus to complete cellular disintegration. The most obvious change shown in sections of the spinal cord was a diminution in size of the anterior horn corresponding to the affected joint. The muscles showed discolouration of the fibres with absence or impairment of the transverse striæ. Dr. Pighini considers that the results of his experiments prove that amyotrophia following shortly after the onset of articular inflammation is of reflex origin, and he thinks that the atrophy is probably due to ischæmia caused by prolonged reflex vascular spasm.

